# Effects of intravenous glucocorticoids on postoperative delirium in adult patients undergoing major surgery: a systematic review and meta-analysis with trial sequential analysis

**DOI:** 10.1186/s12871-023-02359-8

**Published:** 2023-12-06

**Authors:** Chengwei Li, Zheng Zhang, Lin Xu, Xiaojie Lin, Xinyi Sun, Jianjun Li, Penghui Wei

**Affiliations:** 1 Department of Anesthesiology, Qilu Hospital (Qingdao), Cheeloo College of Medicine, Shandong University, Qingdao, 266035 P.R. China; 2 Department of Anesthesiology, Qilu Hospital of Shandong University, Jinan, 250000 P.R. China

**Keywords:** Postoperative delirium, Neuroinflammation, Glucocorticoid, Anesthesia, Meta-analysis

## Abstract

**Background:**

The effects of intravenous glucocorticoids on postoperative delirium (POD) in adult patients undergoing major surgery remain controversial. Therefore, we conducted this meta-analysis to assess whether intravenous glucocorticoids can decrease POD incidence in the entire adult population undergoing major surgery and its association with patients age, type of surgery, and type of glucocorticoid.

**Methods:**

We searched the relevant literature published before November 3, 2023, through Cochrane Library, PubMed, Embase, and Web of Science. The primary outcome was POD incidence. The risk ratio for the primary outcome was calculated using the Mantel–Haenszel method. The secondary outcomes included 30-day mortality, length of hospital stay, ICU duration, mechanical ventilation duration, and occurrence of glucocorticoid-related adverse effects (e.g., infection and hyperglycemia). This meta-analysis was registered in PROSPERO: CRD42022345997.

**Results:**

We included eight randomized controlled studies involving 8972 patients. For the entire adult population undergoing major surgery, intravenous glucocorticoids reduced the POD incidence (risk ratio = 0.704, 95% confidence interval, 0.519–0.955; *P* = 0.024). However, subgroups defined by type of surgery showed differential effects of glucocorticoids on POD. Intravenous glucocorticoids can not reduce POD incidence in adult patients undergoing cardiac surgery (risk ratio = 0.961, 95% confidence interval, 0.769–1.202; *P* = 0.728), with firm evidence from trial sequential analysis. However, in major non-cardiac surgery, perioperative intravenous glucocorticoid reduced the incidence of POD (risk ratio = 0.491, 95% confidence interval, 0.338–0.714; *P* < 0.001), which warrants further studies due to inconclusive evidence by trial sequence analysis. In addition, the use of glucocorticoids may reduce the mechanical ventilation time (weighted mean difference, -1.350; 95% confidence interval, -1.846 to -0.854; *P* < 0.001) and ICU duration (weighted mean difference = -7.866; 95% confidence interval, -15.620 to -0.112; *P* = 0.047).

**Conclusions:**

For the entire adult population undergoing major surgery, glucocorticoids reduced the POD incidence. However, the effects of glucocorticoids on POD appear to vary according to the type of surgery. In patients receiving major non-cardiac surgery, glucocorticoid may be an attractive drug in the prevention of POD, and further studies are needed to draw a definitive conclusion. In cardiac surgery, intravenous glucocorticoids have no such effect.

**Supplementary Information:**

The online version contains supplementary material available at 10.1186/s12871-023-02359-8.

## Introduction

Postoperative delirium (POD) is an acute and fluctuating disturbance in awareness and attention after surgery. It is considered a common postoperative neurological complication in elderly patients which is associated with poor quality of life and a 30-day mortality rate of approximately 7–10% [1–3]. POD can develop following major procedures, especially cardiac and major non-cardiac surgeries [4, 5]. It commonly occurs between postoperative days 2–5, with an incidence of as high as 70% in high-risk major non-cardiac surgery patients [6]. Recently, animal and human studies on POD have been increasing; however, the pathogenesis and effective prevention of POD remain unclear.

The strongest risk factors for POD include type of surgery, advanced age (> 65 years), and dementia [7]. In recent years, several mechanisms have been proposed to explain the pathogenesis of POD, and neuroinflammation resulting from anesthesia and surgical trauma-activated peripheral immune cells across the blood–brain barrier is considered to play a prominent role in neuronal dysfunction and POD [8–10]; therefore, inhibiting inflammation may theoretically decrease the risk of POD. Glucocorticoids have powerful anti-inflammatory effects mediated via various mechanisms [11, 12]. Some studies, including those that involve cardiac or major non-cardiac surgeries, investigated the effects of intravenous glucocorticoids on the incidence of POD, with results that varied based on the type of surgery. Therefore, we conducted this meta-analysis to explore the effects of glucocorticoids in entire adult major surgical population and assess the effect of type of surgery, type of glucocorticoid and age of patients on POD incidence, with the use of trial sequential analysis to assess the certainty of the evidence.

## Methods

This meta-analysis was conducted following the Preferred Reporting Items for Systematic Reviews and Meta-Analysis (PRISMA) and Assessing the Methodological quality of Systematic Reviews (AMSTAR-2) [13, 14]. This meta-analysis was registered in PROSPERO: CRD42022345997.

### Search strategy and selection criteria

We searched the PubMed, Embase, Cochrane Library, and Web of Science databases for relevant articles published before November 3, 2023. We included studies that met the following criteria: 1. Patients: adult patients receiving major surgery; 2. Intervention: intravenous glucocorticoids; 3. Comparison: placebo; 4. Outcomes: incidence of postoperative delirium in the glucocorticoid and control groups; 5. Study design: randomized controlled trials. The language of the articles was limited to English. We excluded studies that involved patients aged < 18 years, patients undergoing minor surgery, animals, no available assessment tools for POD, or the use of non-intravenous glucocorticoids. Additionally, we excluded studies with unavailable full text or data. We used keywords such as "delirium," "glucocorticoids," and "randomized controlled trials" in our search, and the complete search strategy is given in the eAppendix in the Supplement. After removing duplicate studies, two authors (C.L. and Z.Z.) independently conducted a preliminary screening by reading the titles and abstracts and removed the literature that did not meet the inclusion criteria. Then we retrieved the full text of the remaining studies. Finally, we selected the studies that met the inclusion criteria by reading the full text. Any disagreements were resolved through discussions with other two authors (J.L. and P.W.).

### Assessment of risk of bias and data extraction

C.L. and Z.Z. independently assessed the quality of studies using the Cochrane Risk of Bias tool [15]. In addition, C.L. and Z.Z. used a predesigned table to independently extract the required data, including the first author; year of publication; type of surgery; age of patients; number of patients in the control and intervention groups; type, dose, and timing of glucocorticoids; POD assessment tool; time of POD assessment; occurrence of POD; length of hospital stay; mechanical ventilation duration; ICU duration; 30-day mortality; occurrence of hyperglycemia and occurrence of infection. Any disagreements were resolved through discussions with other two authors (J.L. and P.W.).

### Outcomes

The primary outcome was the incidence of POD. The secondary outcomes included 30-day mortality, length of hospital stay, ICU duration, mechanical ventilation duration, and occurrence of glucocorticoid-related adverse effects (e.g., infection and hyperglycemia).

### Data analysis

We used the Stata14 software for data analysis. We used the Mantel–Haenszel method to calculate risk ratios (RRs) and 95% confidence intervals (CIs) for dichotomous data (POD, infection, 30-day death, hyperglycemia). Meanwhile, we used the inverse-variance method to calculate the weighted mean differences (WMDs) and 95% CIs for continuous data (ICU duration, mechanical ventilation duration, and length of hospital stay). In our data analysis, data expressed as medians (interquartile range) were converted to means ± standard deviations [16]. The chi-square test was performed, and the I^2^ statistic was calculated to assess the heterogeneity of the studies. When I^2^ > 40% or *P* < 0.1, a random effects model was used; otherwise, a fixed effects model was used. Forest plots will be made to show the results of syntheses. We planned to perform subgroup analysis according to the type of surgery, the type of glucocorticoid, and the age of patients. We also plotted L'Abbe plot and garbraith plot to assess heterogeneity.

We performed sensitivity analysis by changing the effect size.

### Assessment of publication bias and quality of evidence

If the number is greater than 10, a funnel plot was used to detect publication bias. Grading of Recommendations, Assessment, Development and Evaluations (GRADE) [17] and the GRADE profiler software were used to assess the confidence of evidence for each outcome.

### Trial sequential analysis

We conducted trial sequential analysis (TSA) for the primary outcome to verify whether the results obtained from the meta-analysis were conclusive [18]. A type I error of 5% and a power of 80% were set, and relative risk reduction was defined as 20%. Incidence in control arm was calculated from the incidence in control group of all included studies. We constructed the trial sequential monitoring boundary (TSMB) and futility boundary (FB), and calculated the required information size (RIS). The evidence may be reliable and conclusive when the included sample size reached the RIS, or when the Z curve crossed the TSMB or FB. TSA was performed using the TSA viewer version 0.9.5.10 Beta (www.ctu.dk/tsa).

## Results

### Study selection

**Fig. 1:**
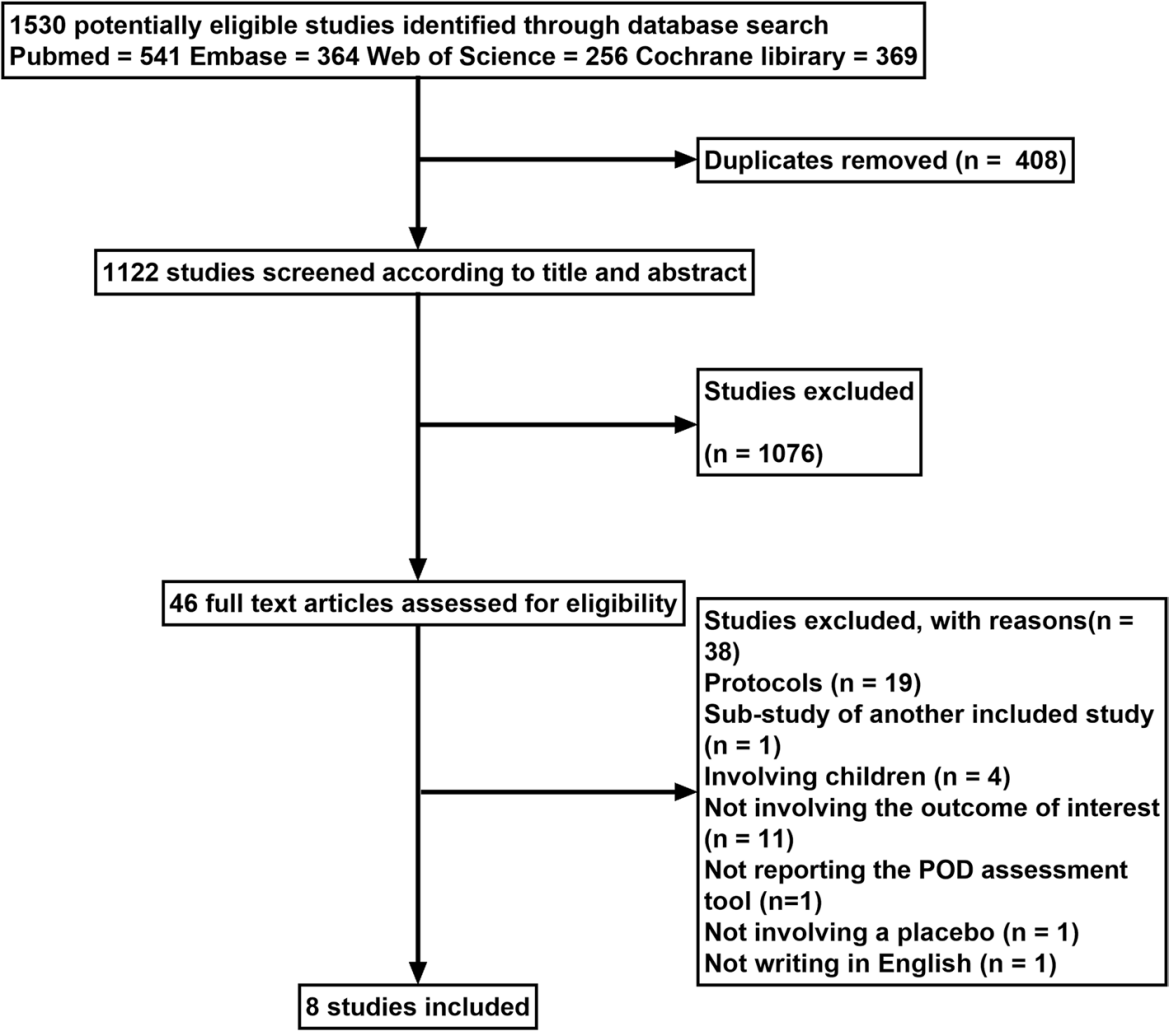
Flow chart of the study selection process

A flowchart of the study selection process is presented in Fig. 1. A total of 1530 studies were identified in the initial search, and after removing 408 duplicates, 1122 articles remained. After screening the titles and abstracts, 1076 studies were additionally removed. The remaining 46 studies were screened by reading the full text, and 38 articles were further excluded (19 protocols, 1 sub-study of another included study, 4 involving children, 11 not reporting the outcome of interest, 1 not reporting the POD assessment tool, 1 not writing in English and 1 not involving a placebo). Finally, eight studies were included in the analysis.

### Study characteristics

The eight randomized controlled trials (RCTs) included 8972 patients [19–26], of whom 4484 patients received glucocorticoids, and 4488 patients received saline. Methylprednisolone was used as the intervention in three of the studies [19, 21, 22], and dexamethasone was used in four studies [20, 23, 25, 26], and one study used hydrocortisone [24]. The dose of the glucocorticoids was inconsistent. Two of the studies involved patients undergoing hip fracture surgery [19, 20], one involved geriatric intertrochanteric fracture patients with internal fixation surgery [26], one involved patients with gastrointestinal surgery [21], and four involved patients with cardiac surgery [22–25]. For the assessment of POD, the evaluation method and assessment time were different. Finally, four of the studies revealed that intravenous glucocorticoids reduced the incidence of POD [19, 21, 25, 26], whereas four concluded that intravenous glucocorticoids cannot prevent POD [20, 22–24]. The characteristics of included studies are shown in Table 1, and summary of outcomes we need in each study is shown in Table 2.

**Table 1:** Characteristics of the patients included in the study

First Author	Year	Type of Surgery	Glucocorticoids, Dose	Control	Number of Patients I/C	Age Mean (± SD) or Median (Interquartile Range) I/C	Assessment Methods, Time
Clemmesen	2018	Hip fracture surgery	MET, 125 mg	NS	59/58	79 (± 8) / 81 (± 9)	CAM-S, POD1-3
Hauer	2012	Cardiac surgery	HYD, 100 mg over 10 min before induction of anesthesia, and 10 mg/h on 24 h (POD1), 5 mg/h on POD2, 3*20 mg on POD3, 3*10 mg on POD4	NS	56/55	69.3 (± 8.9) / 68.0 (± 8.3)	DSM-IV, POD1
Huang	2023	Internal fixation surgery for geriatric intertrochanteric fracture	DEX, 10 mg in 30 min before being sent to the operating room	NS	80/80	84.5 (79.0–89.0) / 85.0 (79.8–90.2)	Nu-DESC and MDAS, POD 1–5
Kluger	2021	Hip fracture surgery	DEX, 20 mg before surgery	NS	40/39	81.4 (± 7.2) / 81.4 (± 8.9)	4AT, POD1-3
Mardani	2013	Cardiac surgery	DEX, 8 mg before surgery, 8 mg every 8 h for the first three post-operative days	NS	43/50	64.55 (± 11.10) / 60.04 (± 12.77)	MMSE, PROD and POD1-3
Sauër	2014	Cardiac surgery	DEX, 1 mg/kg (maximum 100 mg)	NS	367/370	67 (± 12) / 66 (± 12)	CAM-ICU, CAM POD1-4
Whitlock	2015	Cardiac surgery	MET, 250 mg at anaesthetic induction and 250 mg at initiation of CPB	NS	3755/3752	67.5 (± 13.6) / 67.3 (± 13.8)	CAM, POD3
Xiang	2022	Gastrointestinal surgery	MET, 2 mg/kg before surgery	NS	84/84	71 (68–74) / 70 (68–73)	CAM, CAM-S, POD1-5

*I* intervention group, *C* control group, *SD* standard deviation, *MET* methylprednisolone, *NS* normal saline, *CAM-S* confusion assessment method- severity, *POD* postoperative day, *HYD* hydrocortisone, *DSM-IV* diagnostic and statistical manual of mental disorders, fourth revision, *DEX* dexamethasone, *Nu-DESC* the nursing delirium screening scale, *MDAS* the memorial delirium assessment scale, *4AT* the 4 ‘A’s test, *MMSE* minimum mental state examination, *PROD* pre-operative day, *CAM-ICU* confusion assessment method of intensive care unit, *CAM* confusion assessment method, *CPB* cardiopulmonary bypass

**Table 2:** Summary of outcomes in each study

First Author	Incidence of POD I vs C	Infection I vs C	30-Day Mortality I vs C	Length of Hospital Stay Median (Interquartile Range) or Median (Interquartile Range [range]) or Mean ± SD I / C	Blood Glucose Mean ± SD (Interquartile Range) or Median (Interquartile Range [range]) or Mean ± SD I vs C	ICU Duration	Mechanical Ventilation Duration
Clemmesen	10/59 vs 19/58	23/59 vs 32/58	4/59 vs 4/58	Length of postoperative inpatient stay; 8 (6–12[2–35]) days / 9 (6–12[4–46]) days	Not reported	Not reported	Not reported
Hauer	7/56 vs 6/55	Not reported	Not reported	Not reported	Not reported	38.3 ± 31.7 h vs 68.4 ± 49.9 h	17.1 ± 12.0 h vs 21.0 ± 17.0 h
Huang	9/80 vs 21/80	27/80 vs 35/80	Not reported	Not reported	Hyperglycemia 21/80 vs 13/80 Maximum glucose 9.5 [7.3–12.4] mmol/L vs 7.9 [6.5–12.8] mmol/L	Not reported	Not reported
Kluger	6/40 vs 9/39	8/40 vs 3/39	0/40 vs 1/39	16 (5–23 [2–97]) days / 15 (7–25 [3–48]) days	Hyperglycemia 6/40 vs 4/39	Not reported	Not reported
Mardani	4/43 vs 13/50	3/43 vs 2/50	Not reported	12.93 ± 1.03 days vs 13.64 ± 1.75 days	Mean postoperative blood glucose 245 ± 68 mg/dl vs 212 ± 45 mg/dl	2.86 ± 1.3 days vs 3.68 ± 1.33 days	9.18 ± 2.40 h vs 10.56 ± 3.86 h
Sauër	52/367 vs 55/370	Not reported	Not reported	Not reported	Not reported	23 (20–24) h vs 22 (20–24) h	8 (5–10) h vs 8 (6–11) h
Whitlock	295/3755 vs 289/3752	465/3755 vs 493/3752	154/3755 vs 177/3752	9.0 (7.0–13.0) days vs 9.0 (7.0–13.0) days	Peak blood glucose 12.7 ± 7.2 mmol/L vs 12.1 ± 18.7 mmol/L	46.0 (23.0–90.0) h vs 47.0 (24.0–91.0) h	Not reported
Xiang	9/84 vs 20/84	2/84 vs 1/84	0/84 vs 1/84	10 (8–12) days / 10 (8–13) days	Not reported	Not reported	Not reported

*POD* postoperative delirium, *I* intervention group, *C* control group, *SD* Standard Deviation, *ICU* intensive care unit

### Risk of bias

**Fig. 2:**
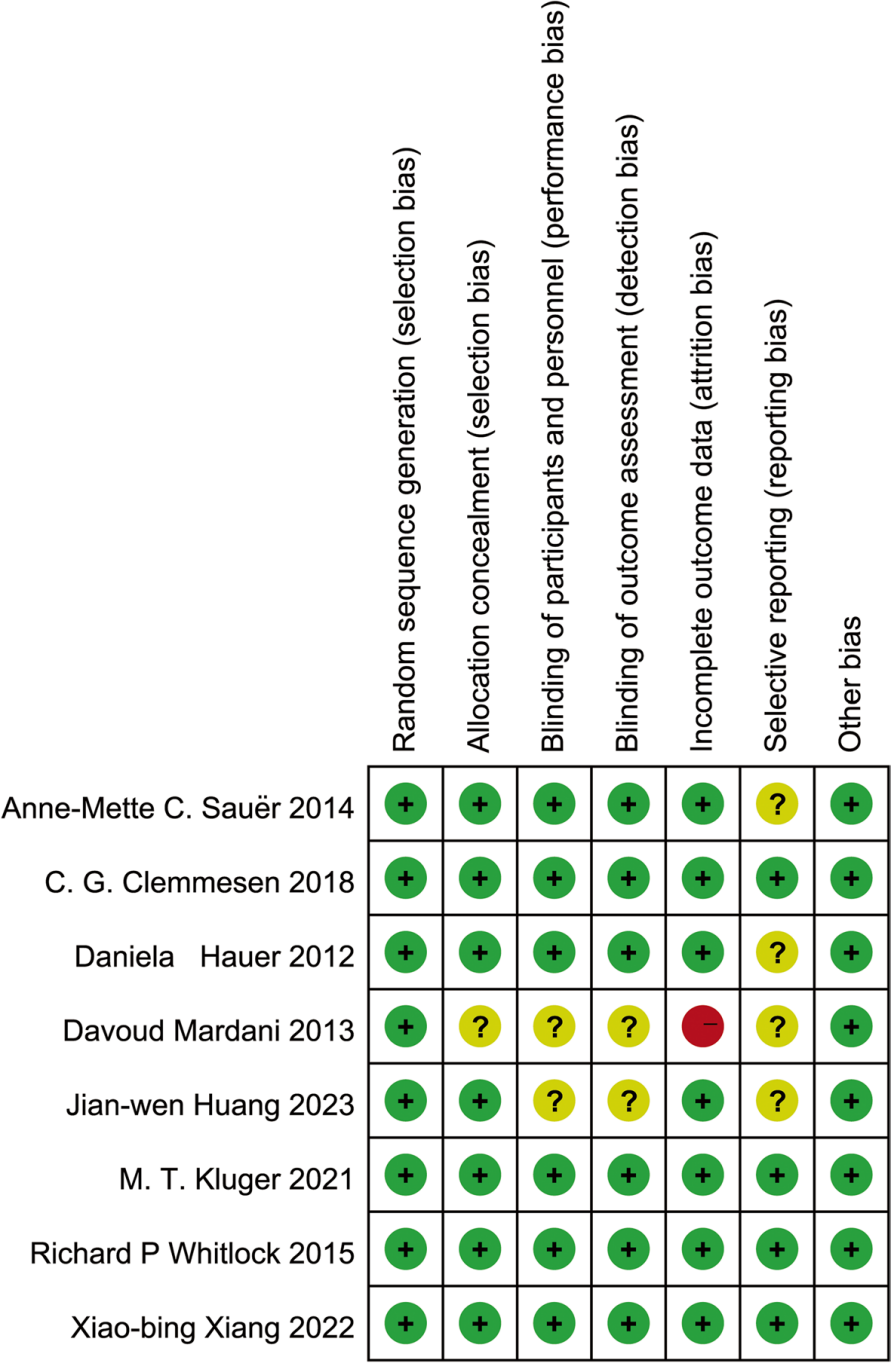
Summary of risk of bias: review authors’ judgments on the risk of bias for each study according to the Cochrane Risk of Bias Methods: ( +), low risk of bias; (?), unclear risk of bias; ( −), high risk of bias

Of the eight included studies, the study by Mardani et al. [25] was considered likely to have a high risk of attrition bias due to incomplete outcome data, and we defined four studies as a low risk of bias. Figure 2 shows the risk of bias for each study.

### Quality of the evidence

**Fig. 3:**
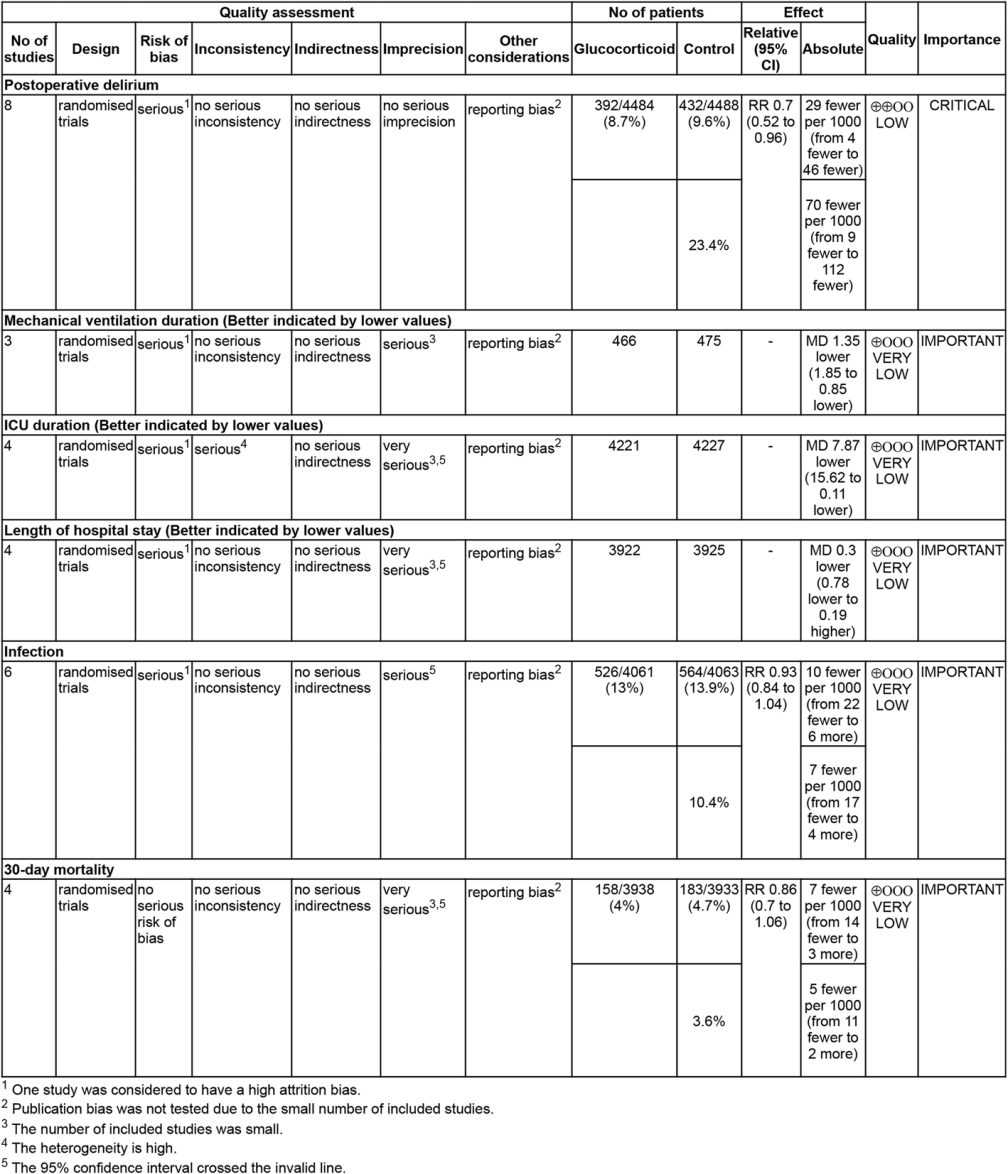
Grading of recommendations, assessment, development, and evaluations evidence profile

According to the GRADE, the quality of evidence for POD was considered “low”, and the quality of evidence for other outcomes was considered “very low” (Fig. 3).

### Primary outcome: POD incidence

**Fig. 4:**
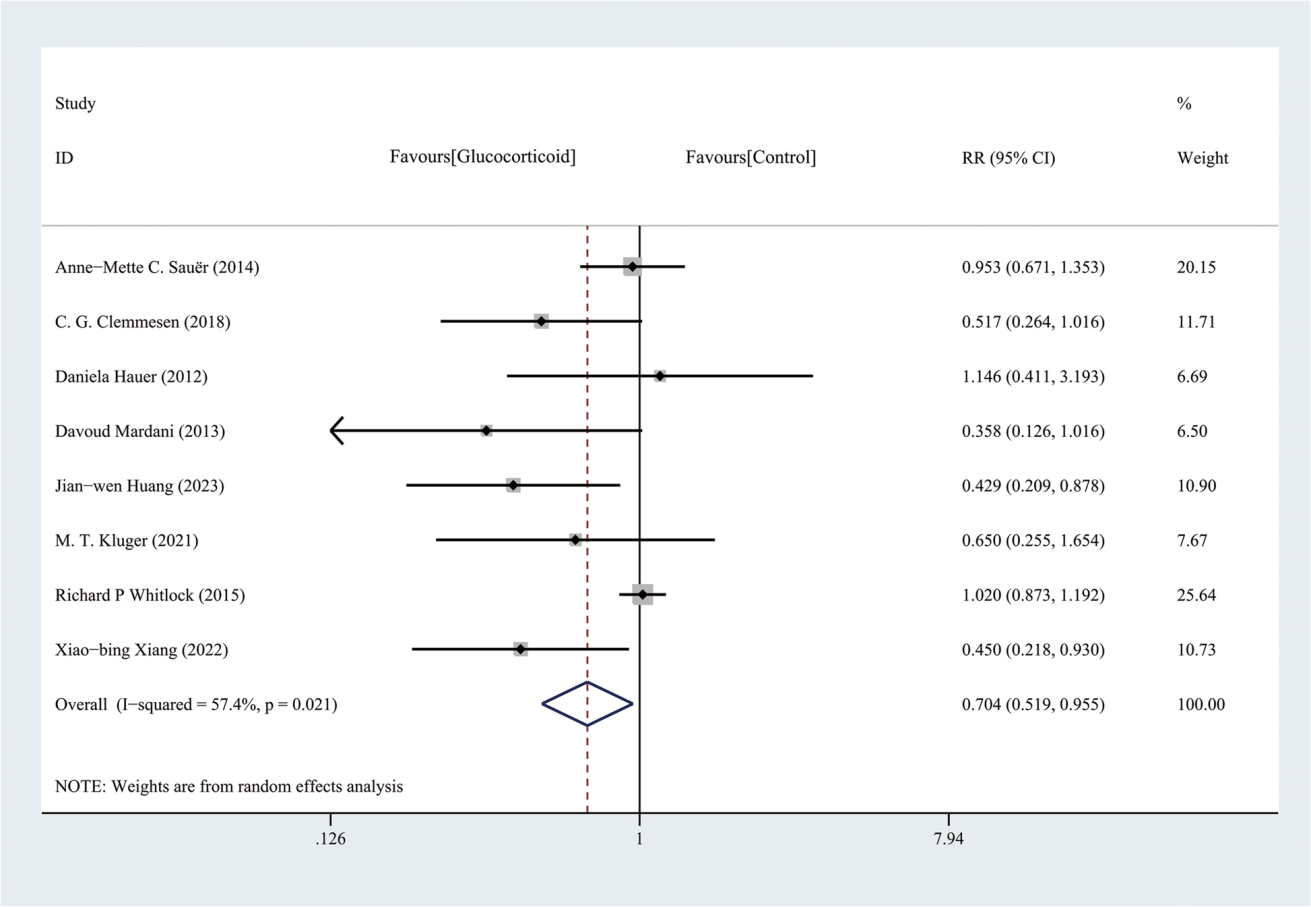
Forest plot of the incidence of postoperative delirium in elderly patients undergoing major surgery using a random-effects model: glucocorticoids vs. control

**Fig. 5:**
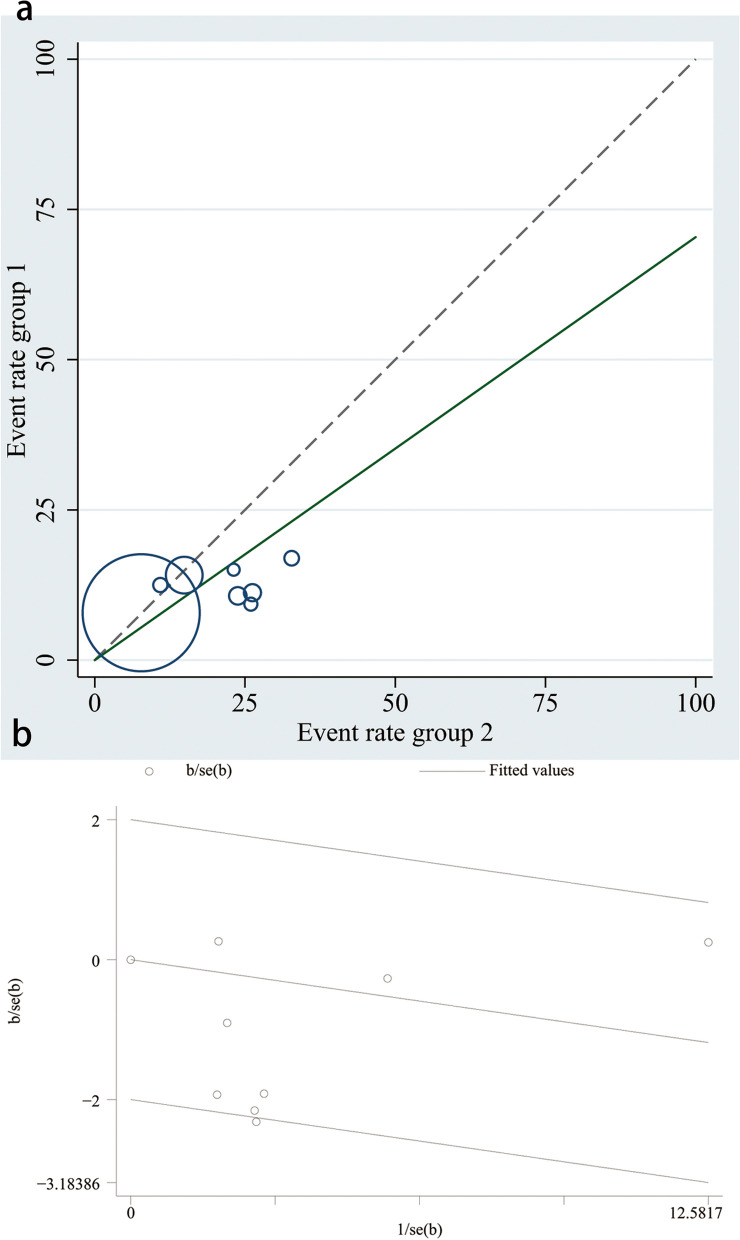
**a **L'Abbe plot. **b **Garbraith plot

**Fig. 6:**
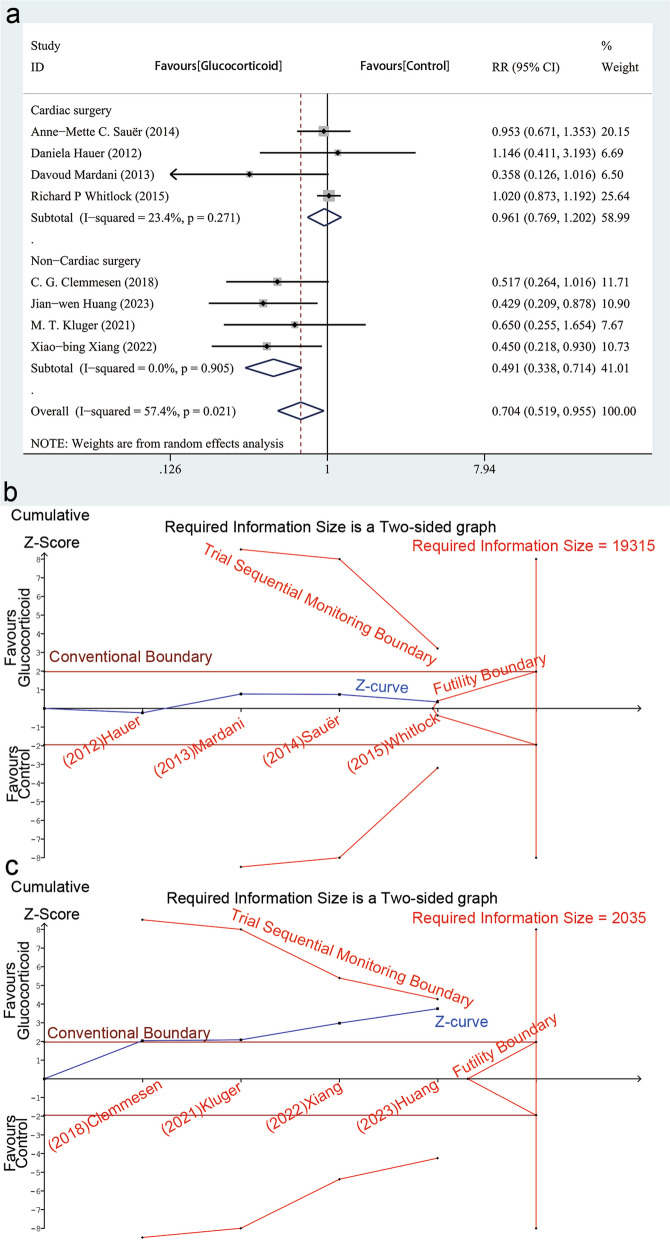
**a **Subgroup analysis according to the type of surgery. **b **Trial sequential analysis for outcome of postoperative delirium in patients undergoing cardiac surgery: α = 5% (two-sided) and β = 20% (power, 80%), incidence in control arm calculated from studies = 8.59%, relative risk reduction = 20%. **c** Trial sequential analysis for outcome of postoperative delirium in patients undergoing major non-cardiac surgery: α = 5% (two-sided) and β = 20% (power, 80%), incidence in control arm calculated from studies 26.44%, relative risk reduction = 20%

Results of meta-analysis showed that intravenous glucocorticoids decreased the incidence of POD in the entire adult population undergoing major surgery (RR, 0.704, 95%CI, 0.519–0.955; *P* = 0.024) (Fig. 4). We conducted a test for heterogeneity (I^2^ = 57.4%; P_heterogeneity_ = 0.021) and plotted L'Abbe plot (Fig. 5a) and garbraith plot (Fig. 5b). It was considered as substantial heterogeneity. Our subgroup analysis according to the type of surgery showed differential effects. In the cardiac surgery group, the results did not show a difference in POD incidence between normal saline and glucocorticoid group (RR, 0.961; 95%CI, 0.769–1.202; *P* = 0.728; I^2^ = 23.4%; P _heterogeneity_ = 0.271); however, the results were not consistent in non-cardiac surgery (RR, 0.491; 95% CI, 0.338–0.714; *P* < 0.001; I^2^ = 0.0%; P_heterogeneity_ = 0.905). The heterogeneity of the two subgroups was reduced; therefore, the type of surgery may be the source of the heterogeneity (Fig. 6a), and the result of meta-regression with type of surgery as a covariate supported this view (eTable 1 in the Supplement). In other words, the effect of glucocorticoid on POD may vary according to the type of surgery, and patients undergoing non-cardiac surgery are likely to benefit more. TSA for cardiac surgery subgroups showed that though not reaching the RIS, the z-curve crossed the FB, providing further evidence that glucocorticoid had no benefit for reducing POD incidence (Fig. 6b). In the subgroup of patients undergoing non-cardiac surgery, the TSA results showed that the cumulative Z-curve crossed the conventional boundary but not the TSMB or the FB and did not reach the RIS, indicating that the information size was insufficient to draw definitive conclusions, and there was a possibility of false positivity for this result, and at this stage of review, 524 patients (only 25.7% of the RIS) were available to detect or reject a relative risk reduction (RRR) of 20% (Fig. 6c). Therefore, more RCTs are needed to verify this result in major non-cardiac surgery.

**Fig. 7:**
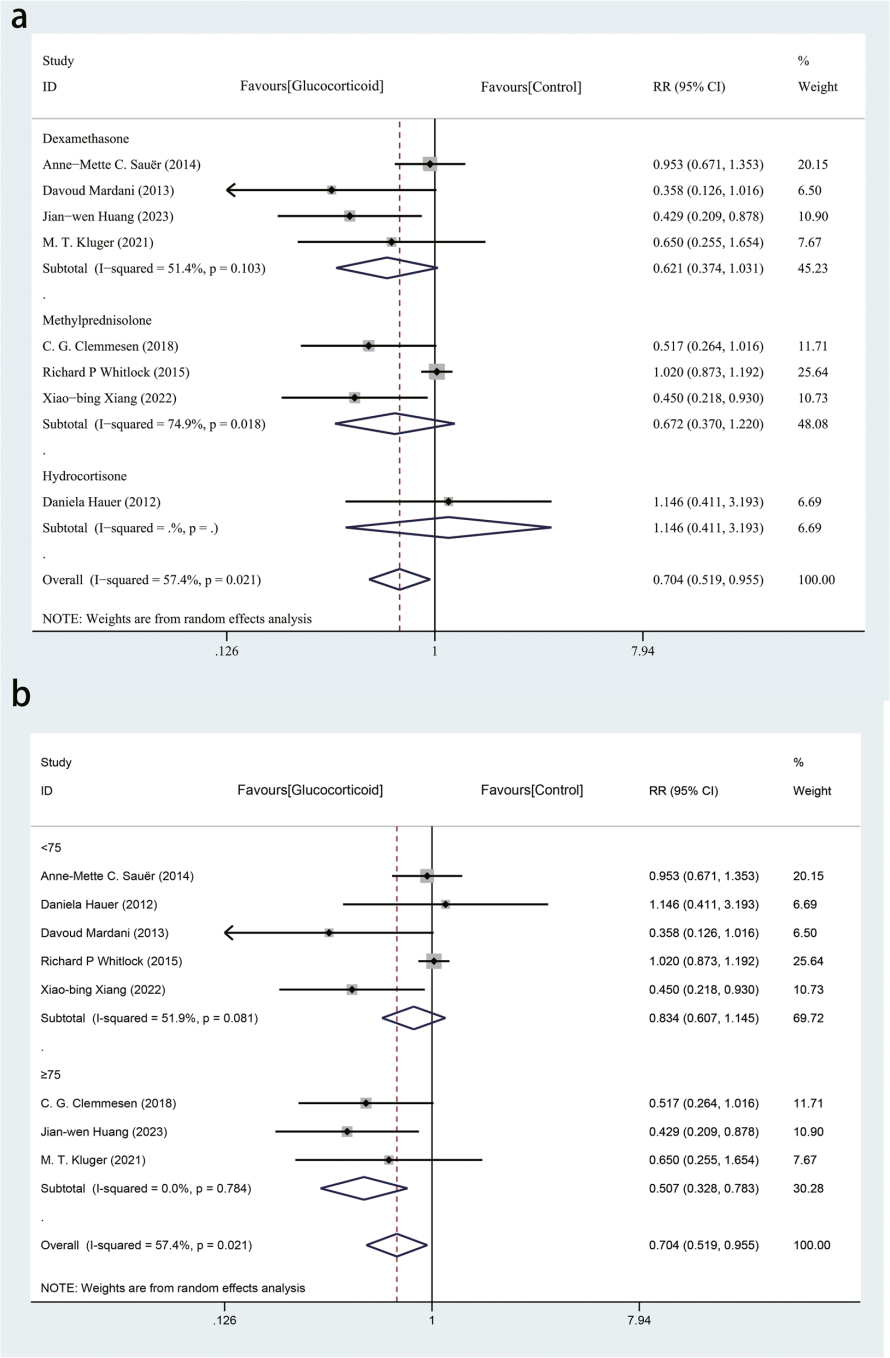
**a **Subgroup analysis based on the type of glucocorticoid. **b** Subgroup analysis based on the age of patients

We also conducted subgroup analysis based on the type of glucocorticoid and found the results were consistent in any of the three subgroups (Fig. 7a). In a subgroup analysis based on the age of patients, we divided studies into two groups (< 75 years and ≥ 75 years), and the results were inconsistent between the < 75 years group (RR, 0.834; 95% CI, 0.607–1.145; *P* = 0.261; I^2^ = 51.9%; P _heterogeneity_ = 0.081) and the ≥ 75 years group (RR, 0.507; 95% CI, 0.328–0.783; *P* = 0.002; I^2^ = 0.0%; P _heterogeneity_ = 0.784) (Fig. 7b). However, the result of meta-regression with age of patients as a covariate not support it as a source of heterogeneity (eTable 2 in the Supplement).

We performed sensitivity analysis by changing the effect size. The results remained consistent when we calculated risk difference and odds ratio as effect sizes (Table 3).

**Table 3:** Sensitivity analysis of the primary outcome

	Risk Ratio (95% confidence interval)	Odds Ratio (95% confidence interval)	Risk Difference (95% confidence interval)
Random-effect Model	0.704 (0.519 to 0.955)	0.648 (0.447 to 0.940)	-0.061(-0.113 to -0.010)

In addition, the study by Whitlock et al. involved 7507 patients, which is quite beyond any other included study, and in order to explore the potential effect of study size on overall outcomes, we removed the study by Whitlock et al. and the conclusion remained consistent. However, when we removed the high-risk study (the study by Mardani et al. with a high attrition bias due to incomplete outcome data), the result showed that the incidence of POD was marginally significant between the two groups (RR, 0.748, 95% CI, 0.556–1.006; *P* = 0.055; I^2^ = 54.9%; P _heterogeneity_ = 0.038). Moreover, when we included only low-risk studies for analysis, the result was inconsistent (RR, 0.680, 95% CI, 0.418–1.108; *P* = 0.121; I^2^ = 65.2%%; P _heterogeneity_ = 0.035), which suggested that the quality of included studies may affect the stability of the conclusion, and more high-quality studies are still needed to provide conclusive evidence in the future.

### Secondary outcomes

**Fig. 8:**
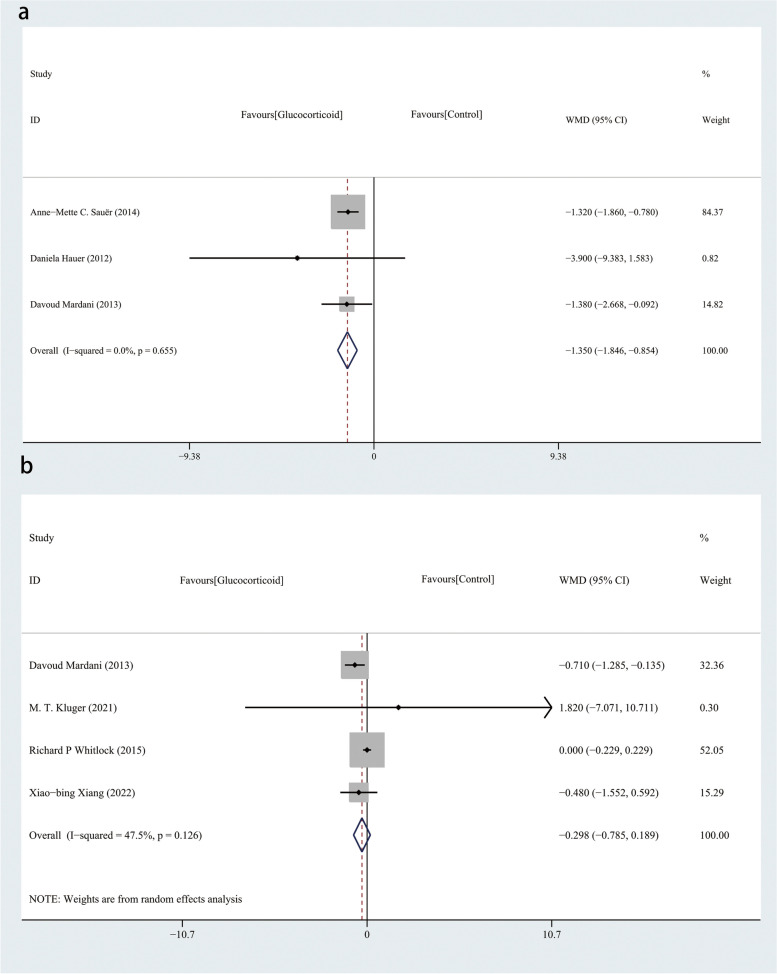
**a **Forest plot for the mechanical ventilation duration with a fixed-effects model: glucocorticoid vs control. **b **Forest plot for the length of hospital stay with a random-effects model: glucocorticoid vs control

**Fig. 9:**
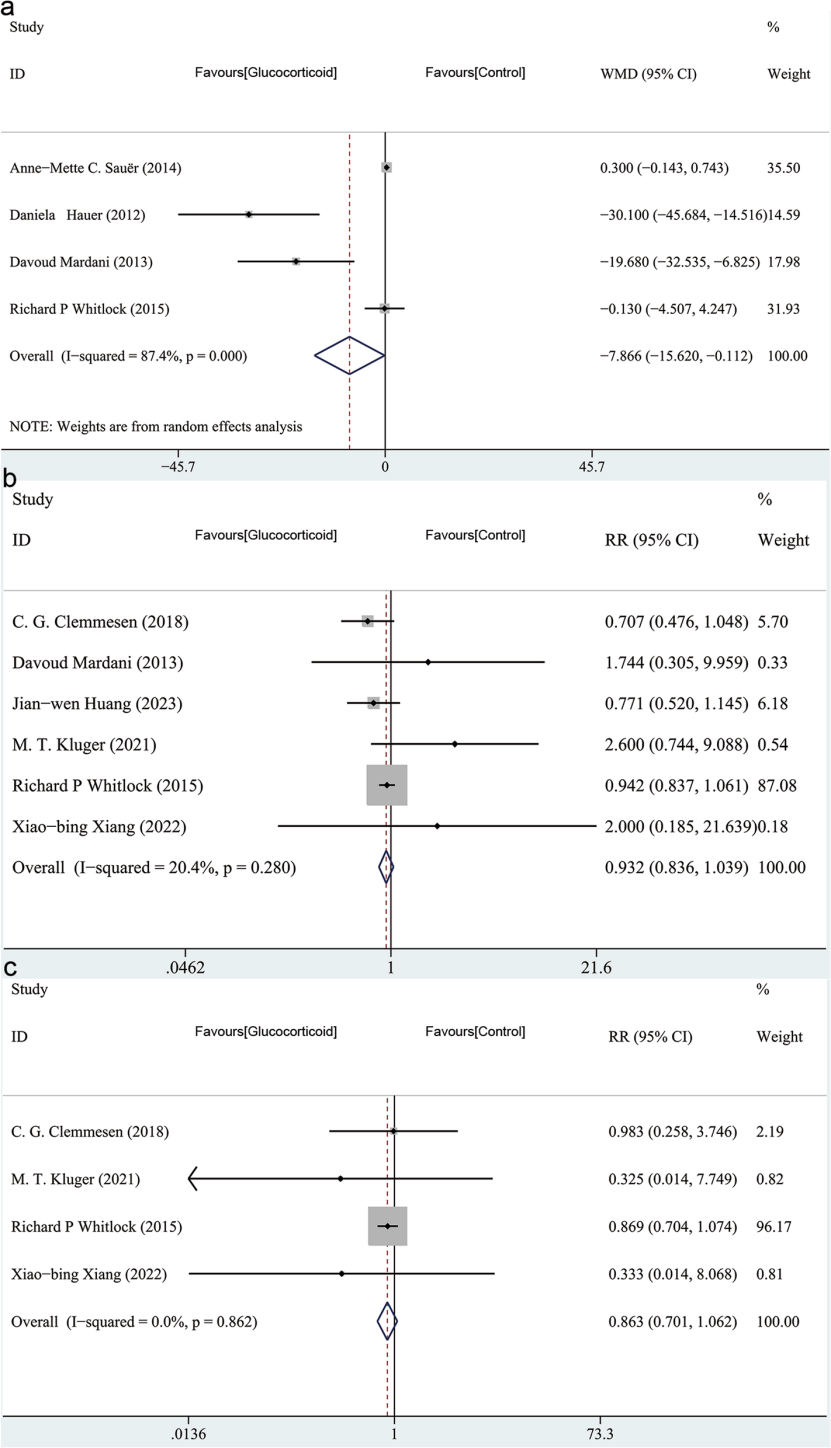
**a **Forest plot for the ICU duration with a random-effects model: glucocorticoid vs control. **b** Forest plot for the infection with a fixed-effects model: glucocorticoid vs control. **c** Forest plot for the 30-day mortality with a fixed-effects model: glucocorticoid vs control

Three of the studies reported the mechanical ventilation duration [23–25]. Intravenous glucocorticoids may have the effect of reducing the mechanical ventilation duration (WMD, -1.350; 95%CI, -1.846 to -0.854; *P* < 0.001; I^2^ = 0.0%; P_heterogeneity_ = 0.655) (Fig. 8a). Four of the studies involved the length of hospital stay as an outcome [20–22, 25]. Compared with the control group, the length of hospital stay was not shorter in the glucocorticoid group (WMD, -0.298; 95%CI, -0.785–0.189; *P* = 0.231; I^2^ = 47.5%; P_heterogeneity_ = 0.126) (Fig. 8b). One study involved the length of postoperative inpatient stay as an outcome instead of the length of hospital stay [19]; therefore, we did not include it in the data analysis. Four studies [22–25] reported ICU duration. Analysis of the data suggests that the use of intravenous glucocorticoids may shorten ICU duration. (WMD = -7.866; 95% CI, -15.620 to -0.112; *P* = 0.047, I^2^ = 87.4%; P_heterogeneity_ < 0.001) (Fig. 9a). In addition, intravenous glucocorticoids did not reduce the infection (RR, 0.932; 95%CI, 0.836–1.039; *P* = 0.203; I^2^ = 20.4%; P_heterogeneity_ = 0.280) (Fig. 9b), and 30-day mortality (RR, 0.863; 95%CI, 0.701–1.062; *P* = 0.164; I^2^ = 0.0%; P_heterogeneity_ = 0.862) (Fig. 9c). As the sample size is not sufficiently large, the credibility of the second outcomes is limited.

Only two study [20, 26] reported hyperglycemia and both found no significant difference. Three studies reported blood glucose values after surgery. One reported that mean postoperative blood glucose was higher in the glucocorticoid group (245 ± 68 mg/dl versus 212 ± 45 mg/dl, *P* = 0.007) [25], and another one reported that peak blood glucose was higher in the glucocorticoid group (12.7 ± 7.2 mmol/L versus 12.1 ± 18.7, *P* = 0.04) [22]; however, one study reported that there is no statistical differences in the maximum glucose between the glucocorticoid group and the control group [26]. Therefore, the safety and optimal dose of intravenous glucocorticoids still requires further investigation.

### Publication bias

Because of the small number of included studies, we did not test for publication bias.

## Discussion

Currently, neuroinflammation is considered to play a prominent role in the pathogenesis of POD in non-cardiac surgery, especially orthopedic surgery, and can be exploited as a modifiable mechanism [27]. Anesthesia and surgery induce peripheral inflammation, causing disruption of the blood–brain barrier, thereby contributing to neuroinflammation and leading to dysfunction of synapses and neurons [28–30]. Glucocorticoids have powerful anti-inflammatory effects, and it has been suggested that perioperative administration of glucocorticoids may benefit significantly by inhibiting inflammation [31]. Therefore, the administration of intravenous glucocorticoids may prevent POD through anti-inflammatory effects in patients undergoing major surgery. A recent meta-analysis concluded that POD cannot be prevented by intravenous glucocorticoids in patients receiving cardiac surgery [32], and we conducted this meta-analysis to explore the effect of glucocorticoids in a general major surgical population, and to further explore the influence of type of surgery, type of glucocorticoid, age of patients on the effects of glucocorticoids. Through subgroup analysis and meta-regression, it was concluded that the type of surgery was the source of heterogeneity. In addition, age of patients and glucocorticoid type were not the source of heterogeneity. Intravenous glucocorticoids could not decrease the incidence of POD in patients undergoing cardiac surgery, with the TSA further improving the credibility of this conclusion. However, in major non-cardiac surgery, glucocorticoids may be beneficial for the prevention of postoperative delirium, with the results of the more robust TSA for cardiac surgery subgroups indicating that the current evidence has insufficient reliability to allow definitive conclusions and that studies are still needed. The reason for this difference may be that there are many risk factors, such as longer surgery time, more blood transfusion, longer mechanical ventilation time and longer critical care unit stay in adult patients undergoing cardiac surgeries, especially cardiopulmonary bypass surgery [33] and an anti-inflammation strategy alone may not be sufficient to prevent POD in these patients.

Other possible sources of heterogeneity included the baseline characteristics of patients, glucocorticoid dose and time of administration, and other intraoperative factors. Most of the studies excluded patients with preoperative cognitive dysfunction, which is considered a risk factor for POD [4] and, therefore, may have an impact on the accuracy of the conclusion. In addition, different diagnostic methods and assessment times between studies may be important sources of heterogeneity. In the study by Mardani et al. [25], MMSE was used to diagnose POD; however, MMSE was not considered very accurate in diagnosing POD [34], and the obtained results may have some influence on the final data analysis. In the study by Huang et al., Nu-DESC was used to screen for delirium and used MDAS for definitive evaluation, which is considered a reasonable method for assessing delirium. In addition, the CAM, CAM-ICU, CAM-S, DSM-IV, and 4AT used in other studies were all considered to have good accuracy and were more suitable for the diagnosis of POD [35–39]. Most of the included studies chose to evaluate 1–3 days after surgery [19, 20, 25], one study evaluated on the third day [22], two evaluated 1-5 days after surgery [21, 26], one study [23] chose to evaluate 1–4 days after surgery, and one study [24] evaluated only on the first day. POD mainly occurs within 24 h to 72 h after surgery [40], and only evaluating on the first day may lead to the failure to include all patients with delirium. This may have influenced the results of the final data analysis. Therefore, POD diagnostic tools and timing should be standardized in future trials.

Elderly patients are the most rapidly increasing group among surgical admissions, and advanced age has been demonstrated as an important risk factor for POD. The median or mean age of patients enrolled in eight studies was all greater than 60. Glucocorticoids are known to exert not only anti-inflammatory, but also immunosuppressive effects by inhibiting cellular immunity [41]. Therefore, the use of glucocorticoids may be associated with a higher risk of infection. However, in this meta-analysis, glucocorticoid administration was not associated with an increased occurrence of postoperative infection. Due to the limited number of studies and patients, the confidence of the results is limited. In addition, data analysis for incidence of infection indicated high heterogeneity, which may be due to differences in type of surgery and follow-up time. Observing the incidence of infection only during the hospitalization period may lead to inaccurate results, and a longer follow-up period may be needed to ensure that no infections are missed. Another common adverse effect of glucocorticoid use is hyperglycemia. Glucocorticoids induce hyperglycemia by increasing insulin resistance and reducing insulin sensitivity [42, 43]. Two included studies did not reveal a difference in hyperglycemia between the glucocorticoid and control groups [20]. However, two other studies reported differences in postoperative blood glucose values between the glucocorticoid group and the control group [22, 25]. Based on the mentioned findings, the safety of perioperative intravenous glucocorticoids is still worthy of attention, and additional studies are needed to explore the optimal dose and timing of perioperative glucocorticoid administration since the adverse effects of glucocorticoids are related to the duration and dose [44].

Our study has some limitations. First, the number of included studies is limited. For patients undergoing major non-cardiac surgery, the sample size did not reach the RIS; therefore, false-positive results might have been obtained, and we did not test for publication bias, which may decrease the reliability of our conclusions. Second, we included only studies on POD, and some studies that involved the secondary outcomes we were interested in may not be included; therefore, the accuracy of the results for the secondary outcomes is limited. Moreover, we restricted language to English, and articles written in other languages were not included, which may have affected the reliability of the outcomes. Finally, although we included adults over 18, the median or mean age of patients enrolled in eight studies was all greater than 60, which affects the extrapolation of conclusions in the youth population.

## Conclusion

The effect of glucocorticoids on postoperative delirium appears to depend on the type of surgery. For patients undergoing cardiac surgery, glucocorticoids cannot decrease POD incidence, while for patients undergoing non-cardiac surgery, glucocorticoids may be potential drugs to reduce POD incidence. However, since TSA in the non-cardiac surgery subgroup showed inconclusive results, more RCTs are warranted. In the future, the effects of glucocorticoids on POD still deserve attention, and future studies should focus on elderly patients undergoing major non-cardiac surgery of various types.

### Supplementary Information

**Additional file 1: eAppendix.** Search Strategy for PubMed, Embase, Cochrane Library, and Web of Science. **eTable 1.** Meta-regression with Type of Surgery as a Covariate. **eTable 2.** Meta-regression with Age of Patients as a Covariate.

## Data Availability

All data generated or analysed during this study are included in this published article [and its supplementary information files].

## Abbreviations

POD: Postoperative delirium
ICU: Intensive care unit
PRISMA: Preferred Reporting Items for Systematic Reviews and Meta-Analysis
AMSTAR-2: A MeaSurement Tool to Assess systematic Reviews -2
RR: Risk ratio
CI: Confidence interval
WMD: Weighted mean difference
GRADE: Grading of Recommendations, Assessment, Development and Evaluations
TSA: Trial sequential analysis
TSMB: Trial sequential monitoring boundary
FB: Futility boundary
RIS: Required information size
RCTs: Randomized controlled trials
RRR: Relative risk reduction
MMSE: Minimum mental state examination
CAM: Confusion assessment method
CAM-S: Confusion assessment method- severity
DSM-IV: Diagnostic and statistical manual of mental disorders, fourth revision
Nu-DESC: The Nursing Delirium Screening Scale
MDAS: The memorial delirium assessment scale
4AT: The 4 ‘A’s test
