# Supplementary material for: Effects of intravenous glucocorticoids on postoperative delirium in adult patients undergoing major surgery: a systematic review and meta-analysis with trial sequential analysis
Source: BMC Anesthesiol. 2023 Dec 6;23:399. doi: 10.1186/s12871-023-02359-8 (PMC10698986; doi:10.1186/s12871-023-02359-8)
Supplement: Supplementary file 1 — Additional file 1: eAppendix. Search Strategy for PubMed, Embase, Cochrane Library, and Web of Science. eTable 1. Meta-regression with Type of Surgery as a Covariate. eTable 2. Meta-regression with Age of Patients as a Covariate. [file 12871_2023_2359_MOESM1_ESM.docx]

Supplement

**Title: Effects of Intravenous Glucocorticoids on Postoperative Delirium in Adult Patients Undergoing Major Surgery: A Systematic Review and Meta-Analysis with Trial Sequential Analysis**

**eAppendix**. Search Strategy for PubMed, Embase, Cochrane Library, and Web of Science.

**eTable 1**. Meta-regression with Type of Surgery as a Covariate.

**eTable 2.** Meta-regression with Age of Patients as a Covariate.

**eAppendix**. Search Strategy for PubMed, PubMed, Embase, Cochrane Library, and Web of Science.

**PubMed**

("Delirium"[MeSH Terms] OR "deliri*"[Title/Abstract] OR "organic brain syndrome"[Title/Abstract] OR "acute encephalopathy"[Title/Abstract] OR "postoperative cognitive impairment"[Title/Abstract] OR "postoperative cognitive dysfunction"[Title/Abstract] OR "acute confusion"[Title/Abstract]) AND (("Glucocorticoids"[MeSH Terms] OR "adrenal cortex hormone*"[Title/Abstract] OR "corti*"[Title/Abstract] OR "glucocorticoid*"[Title/Abstract] OR "steroid*"[Title/Abstract] OR "glucocorticoid effect*"[Title/Abstract] OR "glucorticoid effect*"[Title/Abstract] OR "dexamethasone*"[Title/Abstract] OR "prednisolone*"[Title/Abstract] OR "betamethasone*"[Title/Abstract] OR "fluorometholone*"[Title/Abstract] OR "fluocinolone acetonide"[Title/Abstract] OR "triamcinolone*"[Title/Abstract] OR "methylprednisolone*"[Title/Abstract] OR "hydrocortisone*"[Title/Abstract]) AND (("Randomized Controlled Trial"[Publication Type] OR "Controlled Clinical Trial"[Publication Type] OR "Controlled Clinical Trials as Topic"[MeSH Terms] OR "random*"[Title/Abstract] OR "placebo*"[Title/Abstract] OR "trial"[Title/Abstract] OR "groups"[Title/Abstract] OR "therapeutic use"[MeSH Subheading] OR "drug therapy"[MeSH Subheading]) NOT ("animals"[MeSH Terms] NOT "humans"[MeSH Terms])))

**Embase**

#1 'glucocorticoids'/exp OR glucocorticoids

#2‘adrenal cortex hormone’:ab,ti OR ‘corti*’:ab,ti OR ‘glucocorticoid*’:ab,ti OR ‘glucocorticoid effect*’:ab,ti OR ‘steroid*’:ab,ti OR ‘glucorticoid effect*’:ab,ti OR ‘dexamethasone*’:ab,ti OR ‘prednisolone*’:ab,ti OR ‘betamethasone*’:ab,ti OR ‘fluorometholone*’:ab,ti OR ‘fluocinolone acetonide’:ab,ti OR ‘triamcinolone*’:ab,ti OR ‘methylprednisolone*’:ab,ti OR ‘hydrocortisone*’:ab,ti

#3: #1 OR #2

#4:'delirium'/exp OR delirium

#5:''deliri*':ab,ti OR 'organic brain syndrome':ab,ti OR 'acute encephalopathy':ab,ti OR 'postoperative cognitive dysfunction':ab,ti OR 'acute confusion':ab,ti OR 'postoperative cognitive impairment':ab,ti

#6: #4 OR #5

#7: 'randomized controlled trial': ab,ti OR 'controlled clinical trial':ab,ti OR 'controlled clinical trials as topic':ab,ti OR 'random*':ab,ti OR 'placebo*':ab,ti OR 'rct':ab,ti

#8: #3 AND #6 AND #7

**Cochrane Library**

#1: (adrenal cortex hormone OR corti* OR glucocorticoid* OR glucocorticoid effect* OR steroid* OR glucorticoid effect* OR dexamethasone* OR prednisolone* OR betamethasone* OR fluorometholone* OR fluocinolone acetonide OR triamcinolone* OR methylprednisolone* OR hydrocortisone*):ab,ti,kw

#2: (randomized controlled trial OR controlled clinical trial OR controlled clinical trials as topic OR random* OR placebo* OR RCT):ab,ti,kw

#3: (deliri* OR organic brain syndrome OR acute encephalopathy OR postoperative cognitive dysfunction OR acute confusion OR postoperative cognitive impairment): ab,ti,kw

#4: #1 AND #2 AND #3

**Web of Science**

#1: TS= (adrenal cortex hormone OR corti* OR glucocorticoid* OR glucocorticoid effect* OR steroid* OR glucorticoid effect* OR dexamethasone* OR prednisolone* OR betamethasone* OR fluorometholone* OR fluocinolone acetonide OR triamcinolone* OR methylprednisolone* OR hydrocortisone*)

#2: TS=(deliri* OR "organic brain syndrome" OR "acute encephalopathy" OR "postoperative cognitive dysfunction" OR "acute confusion" OR "postoperative cognitive impairment"）

#3: TS= (randomized controlled trial OR controlled clinical trial OR controlled clinical trials as topic OR random* OR placebo* OR RCT)

#4: #3 AND #2 AND #1

**eTable 1**. Meta-regression with Type of Surgery as a Covariate.

| logRR | Coef. | Std. Err. | t | P>\|t\| | [95% Conf. Interval] | |
| --- | --- | --- | --- | --- | --- | --- |
| Type of surgery | -.7027818 | .2032386 | -3.46 | 0.014 | -1.200089 | -.2054749 |
| _cons | .6951659 | .2378111 | 2.92 | 0.027 | .113263 | 1.277069 |

**eTable 2.** Meta-regression with Age of Patients as a Covariate.

| logRR | Coef. | Std. Err. | t | P>\|t\| | [95% Conf. Interval] | |
| --- | --- | --- | --- | --- | --- | --- |
| Age of patients | -.5355763 | .2923072 | -1.83 | 0.117 | -1.250826 | .1796737 |
| _cons | .3964611 | .3797281 | 1.04 | 0.337 | -.5327001 | 1.325622 |
